# Unilateral “Inactive” Condylar Hyperplasia: New Histological Data

**DOI:** 10.3390/jfmk9040217

**Published:** 2024-11-02

**Authors:** Michele Runci Anastasi, Antonio Centofanti, Angelo Favaloro, Josè Freni, Fabiana Nicita, Giovanna Vermiglio, Giuseppe Pio Anastasi, Piero Cascone

**Affiliations:** 1 Department of Biomedical, Dental Sciences and Morphofunctional Imaging, University of Messina, 98125 Messina, Italy; michele.runci@gmail.com (M.R.A.); antonio.centofanti@unime.it (A.C.); angelo.favaloro@unime.it (A.F.); jose.freni@unime.it (J.F.); fabnicita@unime.it (F.N.); anastasi.giuseppe@unime.it (G.P.A.); 2 Maxillofacial Surgery, UniCamillus School of Medicine, Saint Camillus International University of Health and Medical Sciences, 00131 Rome, Italy; piero.cascone@unicamillus.org

**Keywords:** inactive condylar hyperplasia, ATM, cartilage, histology, immunofluorescence

## Abstract

**Background:** Unilateral condylar hyperplasia (UCH) is characterized by slow progression and enlargement of the condyle, accompanied by elongation of the mandibular body, resulting in facial asymmetry, occlusal disharmony, and joint dysfunction. This condition can be defined as “active” or “inactive”: the active form is characterized by continuous growth and dynamic histologic changes, whereas the inactive form indicates that the growth process has stabilized. Since there are few microscopic studies on the inactive form, this study aims to investigate the histological features and expression of key proteins and bone markers in patients diagnosed with inactive UCH. **Methods:** A total of 15 biopsies from patients aged 28 to 36 years were examined by light microscopy and immunofluorescence for collagen I and II, metalloproteinases 2 (MMP-2) and 9 (MMP-9), receptor activator of nuclear factor- kappa B (RANK), and osteocalcin. **Results:** Our findings indicate that during inactive UCH, the ongoing process is not entirely stopped, with moderate expression of collagen, metalloproteinases, RANK, and osteocalcin, although no cartilage islands are detectable. **Conclusions:** The present study shows that even if these features are moderate when compared to active UCH and without cartilage islands, inactive UCH could be characterized by borderline features that could represent an important trigger-point to possible reactivation, or they could represent a long slow progression that is not “self-limited”.

## 1. Introduction

Unilateral condylar hyperplasia (UCH) is a benign mandibular bone pathology recognized as the most common post-natal anomaly affecting the temporomandibular joint (TMJ) [1,2]. This asymmetry results from abnormal growth of the unilateral mandibular condyle, thus causing the clinical manifestation. UCH has been described as a rare condition, with only a limited number of cases documented in the literature [3,4]. It typically occurs between the ages of 11 and 30, mainly affecting females [5], without any preference for the left or right side. Despite being described for the first time many years ago, the literature has not definitively determined its etiopathogenesis, with hypotheses put forward by some authors ranging from trauma to hormonal alterations or genetic factors [6].

Additionally, it has been described as a self-limiting disorder, meaning that its active growth can stop at any time [1,7]. UCH can be defined as “active” or “inactive” through single photon emission computed tomography (SPECT) evaluation: active UCH is characterized by ongoing growth and dynamic histological changes, while inactive UCH indicates that the growth process has stabilized, with fewer or no ongoing histological changes [8,9,10].

Histologically, during UCH, the bone’s growth line is located deep within the articular cartilage, characterized by islands of cartilage within the bone tissue [11,12]. Cartilage islands in UCH represent a significant histopathological feature reflecting the abnormal growth of the condylar region. Under normal conditions, the mandibular condyle grows through the activity of cartilaginous tissue at the condylar head.

However, in UCH, this process can become disordered, leading to the appearance of cartilage islands abnormally located within the bony tissue of the mandibular condyle, distant from the expected growth cartilage [13,14,15]. The presence of cartilage islands reflects disorganized and proliferative activity in the condylar cartilage. This can contribute to uneven growth and deformity of the condyle, clinically manifesting as facial asymmetry and functional TMJ disorders [16,17,18,19]. Considering the histological characteristics, some authors proposed a classification based on the number and depth of fibrocartilage islands in the bone tissue, categorizing UCH from levels 1 to 4 to denote aggressiveness [20,21,22].

The etiopathogenetic mechanisms of hyperplasia are still not fully understood, primarily because it is rare and challenging to obtain abundant samples for histological and/or molecular investigations. Moreover, few studies have focused on the histological characteristics of inactive condylar hyperplasia. Although UCH may be classified as inactive, its histological features might reveal otherwise, or underlying histological conditions could trigger reactivation [23].

The reactivation of UCH is rare; nevertheless, Wolford et al. characterized a variant of UCH resulting from an expedited growth mechanism. This variant exhibits a vertical growth vector that is purported to manifest at any age and that is not self-limiting [24].

Therefore, it is crucial to accurately distinguish between active and inactive forms to identify the appropriate treatment. Our study aims to investigate, for the first time, the characteristics of inactive condylar hyperplasia through a comprehensive study that systematically describes the expression of key players of UCH, such as collagen, metalloproteinases, bone markers like the receptor activator of nuclear factor- kappa B (RANK), and osteocalcin, alongside classic histological staining techniques.

## 2. Materials and Methods

In this retrospective observational study, 15 intraoperative biopsies were collected from patients aged between 28 and 36, diagnosed with inactive UCH. They provided informed consent and underwent surgical treatment utilizing the “proportional condylectomy” technique.

The study population consisted of patients with a confirmed diagnosis of inactive UCH, as confirmed by negative SPECT results and a condylar discrepancy of less than 10%. Exclusion criteria included the presence of systemic diseases, other causes of mandibular asymmetry (such as functional mandibular laterognathia, neoplastic growths, or trauma-induced condylar changes), pharmacological treatments, positive bone scintigraphy, or a condylar discrepancy greater than 10%.

These samples underwent processing with Optical Microscopy (Nikon, H550L, Tokyo, Japan) [25], including staining with Hematoxylin and Eosin and Masson’s Trichrome staining, as well as Immunofluorescence testing for Collagen I and II, matrix metallopeptidase 2 (MMP-2) and matrix metallopeptidase 9 (MMP-9), RANK, and Osteocalcin.

### 2.1. Surgical Procedures

Clinically and radiologically, the abnormal condylar morphology was superimposed on patients with positive SPECT results. The discrepancy between the two condyles was measured using CT cone beam imaging with the Dolphin software 5.0.16 (Loveland, CO 80537). Model surgery was conducted on a stereolithographic 3D model created from the DICOM file. During the surgery, the portions of the removed condyle were superimposed on similar cases with positive bone scintigraphy.

The patients received general nasotracheal anesthesia. During the patient’s preparation, leaving the ipsilateral temporal region uncovered was advantageous for observing potential contractions of the facial nerve. At the same time, the previously sterilized mouth was partially exposed to evaluate occlusion and midline deviation.

A pretragic preauricular incision was made with a cold blade, extending 1 to 2 cm in the temporal region in an arched course of 308° from back to front and bottom to top, enhancing surgical exposure in this region. Dissection commenced by exposing the superficial temporalis fascia, the deep temporalis fascia, and the zygomatic arch. The presence of yellow fat facilitated the identification of the deep temporalis fascia. Upon reaching the temporal muscle’s deep fascia, a tissue dissection was conducted inferiorly to reveal the zygomatic arch. This safety plan indicates that the frontal branch of the facial nerve traverses the superficial fascia, precisely aligned with the zygomatic arch.

Preserving the tissues was crucial to avoid facial nerve injuries, with tissue retraction using hooks preferred over other retractors. The parotid gland was separated from the perichondrium surrounding the tragus cartilage and the external auditory canal in its entirety, thus necessitating its severance from the temporomandibular joint capsule. It was crucial to avoid damaging the perichondrium encasing the tragal cartilage during the dissection. The mobility of the mandibular condyle was evaluated by manipulating the patient’s mandible.

In this region, the superficial temporal artery and vein were identified and preserved by carefully tying and interrupting them, followed by identifying and maintaining the auriculotemporal nerve. The vascular structures were safeguarded and maintained in the posterior aspect of the parotid area. Following the interruption of the vascular systems, the dissection proceeded by penetrating farther to separate the parotid gland. It is ideal to preserve the auriculotemporal nerve, which often runs parallel and next to the superficial temporal vein.

The surgeon used dissection instruments to locate and expose the temporomandibular joint capsule, which had been previously discovered through palpation. The insertion of the lateral ligament was situated on the lateral pole of the condylar head. The insertion was made with a cold blade, and the disk was gently moved upward to expose the temporomandibular joint’s inferior compartment.

The superior compartment of the temporomandibular joint must not be incised during this surgical technique. The lateral ligament must be retained to facilitate discopexy using an anchor screw. Dunn–Dautrey temporomandibular joint condyle retractors were then inserted to carefully protect the disk and the medial structures of the condyle. To minimize exposure and carefully preserve all the functional temporomandibular joint structures, the condylectomy is performed in slices with piezo-surgery.

During the surgery, the lateral ligament attached to the disk was moved antero-medially. It is necessary to identify it and to hold it firmly with an anchor screw discopexy to complete the surgery. An invitation hole for the screw was made on the postero-lateral face of the condyle, followed by discopexy of the articular disc by suturing the lateral ligament with a resorbable anchor screw. 

**Figure 1:**
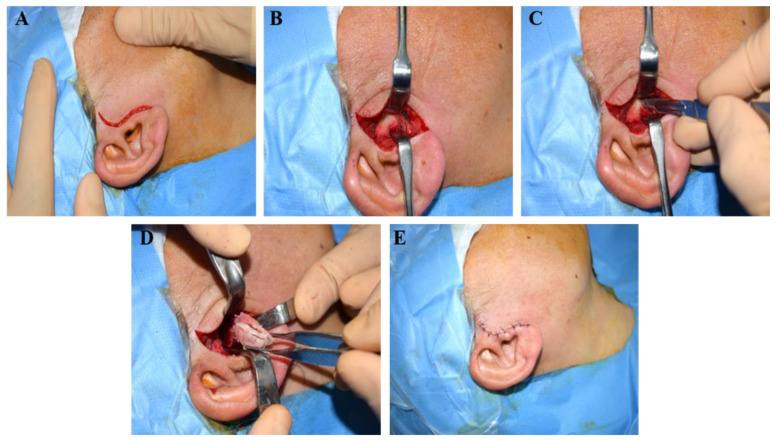
Surgical procedure pictures: (**A**) pretargic preauricular incision; (**B**) exposure of the joint capsule; (**C**) lateral ligament incision; (**D**) sliced condylectomy; (**E**) final sutures.

A significant portion of the continuity of the joint capsule will be restored. Upon completion of these procedures, an arthrocentesis of the superior compartment of the temporomandibular joint was conducted. The upper chamber of the joint was cleansed using isotonic solutions, precisely, 20 mL of lactated Ringer’s solution. This was accomplished by the insertion of two 18-to-20-gauge needles. One needle was placed into the posterior region of the upper compartment, while the second was introduced into the anterior region of the upper compartment. Deeper suturing was conducted utilizing absorbable thread stitches (monofilament or braided 3/0 multifilament). Subsequently, the skin was sutured using 5/0 nylon thread stitches. The surgical procedures are represented in Figure 1A–E.

### 2.2. Sample Preparation for Histology and Immunofluorescence

The samples obtained by surgical operations were preserved overnight in 4% paraformaldehyde in 0.05 M phosphate buffer at 4 °C, dehydrated in ethanol, and decalcified using Technovit 9100 (Heraeus Kulzer GmbH, Wasserburg, Germany) [26,27,28]. Sections of 7 μm thickness were cut with a Leica RM 2125RT microtome (Leica Biosystem, Nussloch, Germany) and collected onto glass slides. The slides were finally deparaffinized by xylene, rehydrated, and used for Hematoxylin–Eosin staining, Masson’s Trichromatic Staining, and Immunofluorescence techniques.

### 2.3. Masson’s Trichromatic Staining 

Masson’s trichromatic is a tricolor staining protocol used in histology to identify collagen tissues, such as muscle and bone, as well as the presence of cells in those tissues. For that purpose, three solutions were previously prepared. The Biebrich scarlet-acid fuchsin solution comprised 90.0 mL of 1% aqueous Biebrich scarlet, 10.0 mL of 1% aqueous acid fuchsin, and 1.0 mL of glacial acetic acid. The phosphomolybdic–phosphotungstic acid solution was made of 5.0 g of phosphomolybdic acid, 5.0 g of phosphotungstic acid, and 200.0 mL of distilled water. The aniline blue solution was made from 2.5 g of aniline blue, 2.0 mL of acetic acid, and 100.0 mL of distilled water. The light green solution comprised 5.0 g of light green dye, 250.0 mL of distilled water, and 2.0 mL of glacial acetic acid. 

Tissues were initially immersed in Bouin’s solution for 1 h at 56 °C. This solution consisted of 75.0 mL of picric acid-saturated aqueous solution, 25.0 mL of 37–40% formalin, and 5.0 mL of glacial acetic acid. After cooling and washing the tissues in running water, samples were rinsed again in distilled water and immersed in the Biebrich scarlet-acid fuchsin solution for 15 min. After another rinse in distilled water, tissues were exposed to a phosphomolybdic and phosphotungstic acid solution for 10–15 min before adding an aniline blue solution. Tissues were immersed in 5% aqueous phosphotungstic acid for 15 min before adding the light green counterstain. After submerging the tissues in aniline blue solution for 5–10 min, samples were rinsed in distilled water and 1% acetic water for 3–5 min. Next, the samples were passed twice through 95% absolute alcohol and then xylene. Finally, samples were mounted in Permount. 

### 2.4. Immunofluorescence Reactions

The slides were treated with 1% bovine serum albumin and 0.3% Triton X-100 (Sigma-Aldrich, St. Louis, MO, USA) in PBS for 30 min at room temperature to obstruct nonspecific binding sites and permeabilize membranes. Finally, the sections were incubated with primary antibody anti Collagen type I, Collagen type II, MMP-2 and MMP-9, RANK, and Osteocalcin. The anti-RANK antibody was detected by Texas-Red conjugated secondary antibody; the other primary antibodies were detected by FITC conjugated secondary antibody. The sections were further investigated, and images were obtained utilizing a Zeiss LSM 5 DUO (Carl Zeiss, Jena, Germany) confocal laser scanning microscope [29]. All photos were digitized at a resolution of 8 bits into an array of 2048 × 2048 pixels. Optical sections of fluorescent specimens were acquired utilizing a helium–neon (HeNe) laser (Carl Zeiss, Jena, Germany) with a wavelength of 543 nm at a scanning speed of 62 s and up to eight averages; sections of 1.50 μm were achieved using a pinhole of 250. Contrast and brightness were determined by analyzing the most luminously labeled pixels and selecting the parameters that facilitated clear visualization of structural features while preserving the maximum pixel intensity (~200). The fluorescence intensity was evaluated through the ImageJ software 1.54k (LOCI, University of Wisconsin). The fluorescence intensity was evaluated over four microscopic fields for each antibody and sample to perform this assessment. The means and standard deviations of the sixty values obtained for each antibody were calculated and shown by graphics.

## 3. Results

### 3.1. Proportional Condilectomy

From a clinical perspective, our patients demonstrate complete facial symmetry recovery. Consequently, with a minimally invasive approach compared to orthognathic surgery, we achieve aesthetic outcomes comparable to those of traditional procedures. The potential complications associated with this surgery include facial nerve injury and other complications that are inherent to all surgical procedures, such as bleeding, infection, and surgical wound dehiscence. The patients included in our study did not experience any of these complications.

### 3.2. Histology

**Figure 2:**
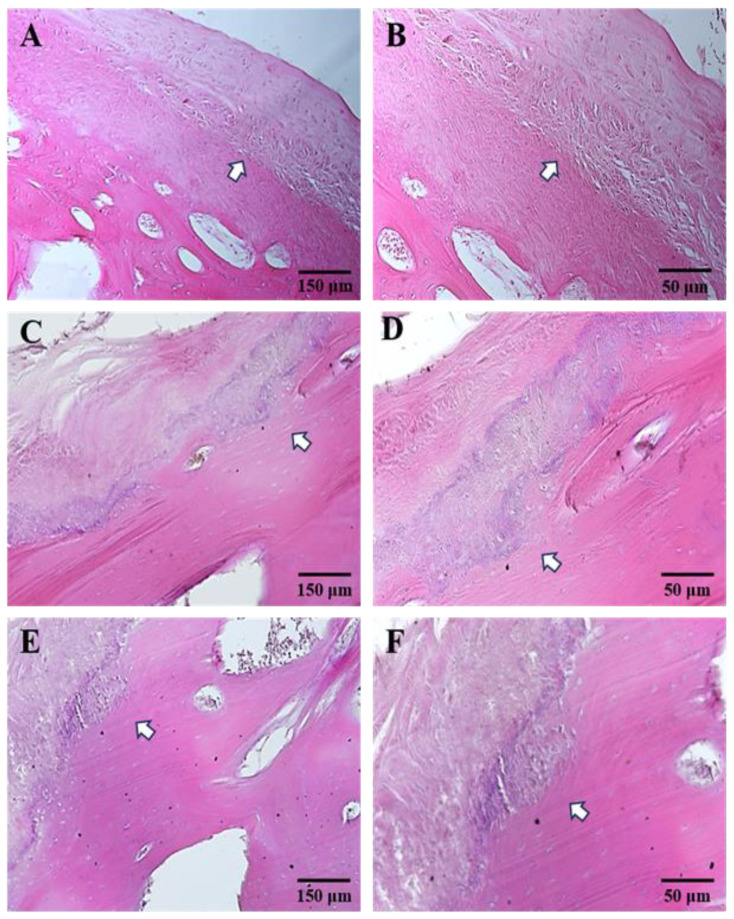
Compound panel of Hematoxylin–Eosin stained sections of UCH (**A**–**F**). Pictures show a thick hyperplastic layer ((**A**,**B**) white arrows) and the existence of an irregular cartilage–bone interface with areas in which the cartilage deepens into the bone tissue without ever detaching in the form of an island ((**C**–**F**) arrows). (**A**,**C**,**E**) 10× magnification; (**B**,**D**,**F**) 20× magnification.

The biopsies from all examined patients exhibited similar pathological characteristics, differing only in the severity of the lesions. Hematoxylin–Eosin staining revealed the four layers of articular cartilage from the outermost to the innermost layers: the fibrous articular layer, the undifferentiated mesenchyme layer, the transitional layer, and the hypertrophic cartilage layer can be distinguished (Figure 2A,B). In all patients, the thickness of the hypertrophic layer appeared significantly increased (Figure 2). In addition, the analysis undertaken with the light microscope indicated that the deepest layer of articular cartilage had invaded the underlying bone tissue (Figure 2C–F). When analyzed in detail, the linear front, which distinguishes the condylar cartilage from the bone tissue, appeared irregular in relationship to the frequent presence of expansion processes with different dimensional and morphological features (Figure 2, white arrows).

**Figure 3:**
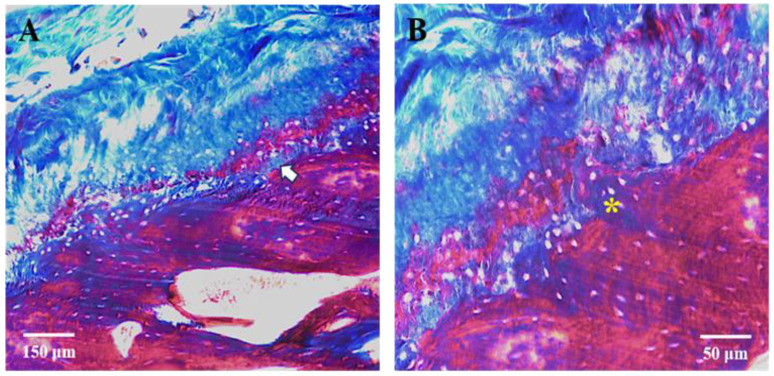
Panel of Masson staining showing expansion processes of the hypertrophying layer in bone tissue ((**A**) white arrows) or infiltration processes of chondrocytes in bone tissue ((**B**) yellow asterisk). It is also possible to observe a double osteochondral border. Magnifications: 10× (**A**); 20× (**B**).

Related to this latter aspect, the use of Masson’s trichrome staining proved very intriguing insofar as if we consider that, with this method, the articular cartilage is highlighted in blue and the bone tissue in red, it was possible to observe a thin, red-colored layer within the blue-colored hypertrophic cell layer on many slides; in other words, it was possible to observe a double osteochondral border (Figure 3A,B). This last histological finding can be explained as a thin layer of bone tissue included in the hypertrophic cell layer during growth. Additionally, it was possible to observe a group of chondrocytes immersed in the collagenic matrix originating from the deeper zone of the hypertrophic layer, which penetrates the underlying bone tissue, indicating an infiltration process. In none of the slides from each of the examined biopsies was there any infiltration of condylar cartilage into the bone tissue as a cartilage island, since the cartilage infiltration is never surrounded by bone tissue.

### 3.3. Immunofluorescence

**Figure 4:**
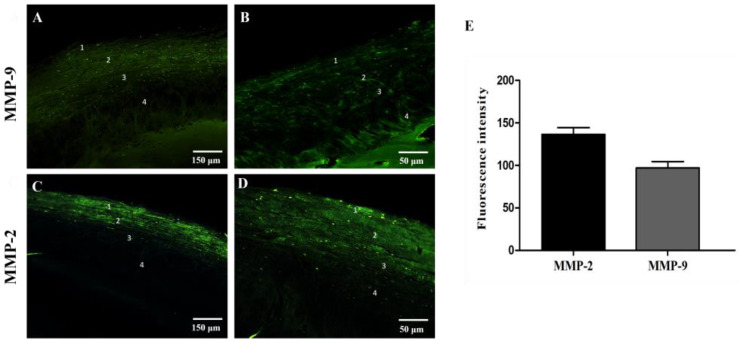
Compound panel of immunofluorescence single localization reactions for MMP-9 (**A**,**B**) and MMP-2 ((**C**,**D**) green channel). It is possible to observe that the MMP-9 staining pattern is more intense in layers 1, 2, and 3 and decreases in layer 4; the same results are observed for MMP-2. (**E**) graphic shows a more intense fluorescence pattern of MMP-2 compared to MMP-9. Magnifications: 10× (**A**,**C**); 20× (**B**,**D**).

The single localization reaction anti-MMPs revealed a detectable immunofluorescence staining pattern for MMP-9 and MMP-2 (Figure 4A–D). Still, the MMP-2 staining pattern was more intense than the MMP-9 one, as revealed by fluorescence intensity analysis (Figure 4E). More specifically, immunostaining for MMP-9 was detected in all four layers of the articular cartilage, which were characterized by different intensities (Figure 4A,B). The fluorescence pattern is shown to be more intense in the superficial zone of the fibrous layer, and it becomes weaker from the deeper zone of the undifferentiated mesenchyme layer to the hyperplastic layer. The immunostaining for MMP-2 exhibited intense immunofluorescence in the deeper regions of the fibrous layer and the undifferentiated mesenchyme layer and weak immunofluorescence in the translational and hyperplastic layers (Figure 4C,D). It was also possible to observe intense immunofluorescence in cellular components of these layers; at higher magnification, we could distinguish between collagen fibers and fibroblast-like cells.

**Figure 5:**
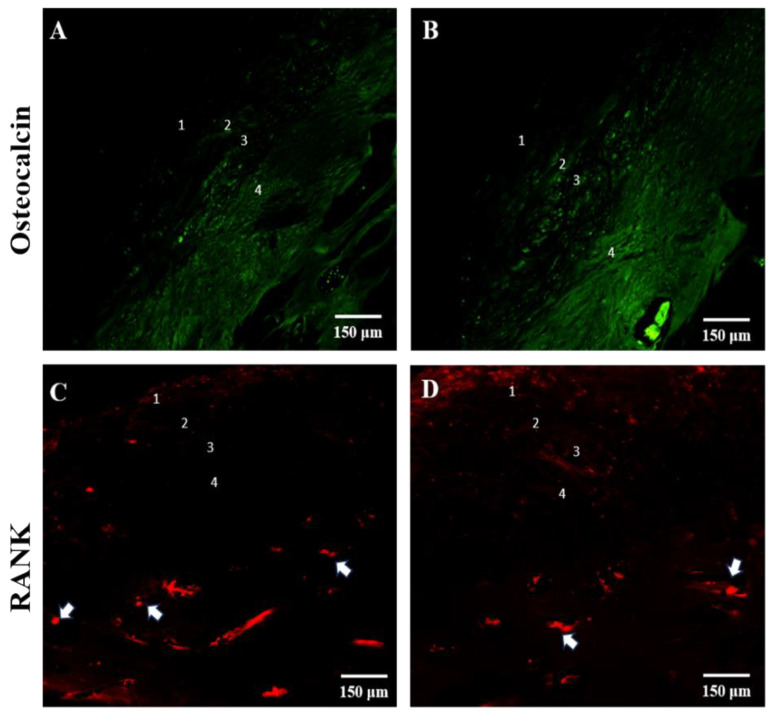
Compound panel of immunofluorescence single localization reactions for osteocalcin ((**A**,**B**) green channel) and RANK ((**C**,**D**) red channel). It is possible to observe that the osteocalcin staining pattern is more intense in layer 4. The RANK staining pattern is well detectable above the cartilage–bone interface and shows osteoclasts within numerous Howship’s lacunae ((**C**,**D**) white arrows. Magnifications 10× (**A**–**D**).

**Figure 6:**
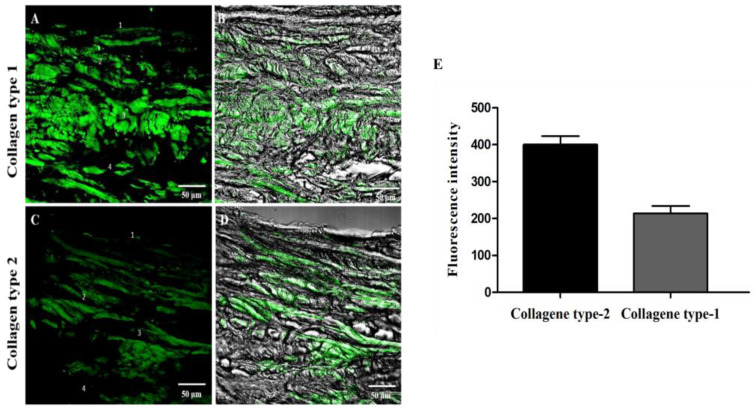
Compound panel of immunofluorescence single localization reactions for Collagen type I ((**A**,**B**) green channel) and Collagen type II ((**C**,**D**) green channel). It is possible to observe that collagen types I and II are expressed along the layers. Still, it is possible to observe a more intense fluorescence pattern for collagen type II compared to collagen type I, as shown by fluorescence intensity analysis (**E**). Magnifications: 20× (**A**–**D**). Transmitted light (**B**,**D**).

Immunofluorescence reaction for osteocalcin revealed weak fluorescence intensity in layers 1 and 2; the fluorescence intensity increased in layer 3 and became more intense in the hyperplastic layer, which is known to be mineralized cartilage that marks the interface between the condylar cartilage and the subchondral bone (Figure 5A,B). The RANK fluorescence pattern is mainly detectable below the cartilage–bone interface, where it is possible to observe a significant number of Howship’s lacunae within which RANK-positive osteoclasts are detectable (Figure 5C,D, white arrows). Immunoreactions for collagen type I and II showed a detectable fluorescence pattern along the layers (Figure 6); in particular, it is possible to observe a more intense fluorescence pattern for collagen type II (Figure 6A,B) when compared to collagen type I (Figure 6C,D), as also shown by fluorescence intensity analysis (Figure 6E).

## 4. Discussion

UCH is a pathological condition characterized by abnormal and excessive growth of the mandibular condyle. This disorder involves abnormal cartilage and bone tissue proliferation within the condyle, driven by several critical histological mechanisms [1,2,3,4]. Proportional condylectomy is a highly effective surgical treatment for UCH [30]. This procedure involves removing part of the enlarged condyle to restore facial symmetry by reducing chin deviation and resolving occlusal issues [31,32]. The excess bone is removed proportionately to maintain the overall condyle shape and function while addressing hyperplasia [33,34]. Outcomes from proportional condylectomy are both predictable and stable as, within 12 months post-surgery, the formation of a neocondyle lodged in the glenoid fossa ensures long-term functional and aesthetic stability [32,33]. However, some patients may experience temporary occlusal changes and alterations in the articular cavity volume [31,33], which tend to normalize over time. Various treatment protocols are used, with some surgeons opting for proportional condylectomy as a standalone procedure [35] while others combine it with orthognathic surgery or orthodontic treatment [36].

It is possible to identify the metabolic state of UCH in active and inactive forms [8,9,10]. The active form is characterized by dynamic histological changes that lead to condylar growth, while the inactive form doesn’t show an ongoing growth process. It is essential to distinguish between these two forms to choose the best therapeutic treatment. Diagnosis of UCH is performed based on radiological and clinical evaluation, and histological characterization is made only if the surgical intervention has been programmed based on the active and inactive diagnosis. In this way, several histological features can be missed in cases of inactive UCH that are treated without surgery. Despite being classified as inactive, histological features might reveal otherwise, or underlying histological conditions could trigger reactivation. Reactivation of UCH is an uncommon phenomenon, but Wolford described a type of UCH caused by accelerated growth of the expected growth mechanism, essentially with a vertical growth vector that is purported to be present at any age and is not self-limited [23,24].

Due to the lack of literature, the present report aimed to perform a comprehensive study that systematically describes the histological features of inactive UCH together with the evaluation of the expression of key components such as collagen, MMPs, and bone markers like RANK and osteocalcin. Our Hematoxylin–Eosin and Masson’s results showed: (1) a significantly increased hypertrophic layer, (2) the alteration of the cartilage–bone interface with the deepest layer of articular cartilage invading the underlying bone tissue by expansion processes with different dimensional and morphological features, (3) the presence of a double osteochondral border. Indeed, in all our patients, the osteochondral border was irregular in appearance in two morphological aspects. One aspect is represented by numerous expansion processes, with different morphological features present in the osteochondral border, and the other is defined by frequent interruptions due to a group of chondrocytes that leak into the underlying bone tissue. It is possible to deduce that these two structural findings can be chronologically linked. In other words, it is likely that, initially, the proliferation activity of chondrocytes induces the penetration of a group of chondrocytes, immersed in their collagenic matrix, in the underlying bone tissue, as already demonstrated [37]. Progressively, the fusion of these chondrocyte groups becomes an expansion process, moving towards the osteochondral border in the bone tissue. With the gradual articular cartilage growth downwards, the bone tissue responds with an ossification procedure upwards that occupies the hypertrophying cartilage, inducing the elongation of the condylar process. If this modality of condylar elongation is suitable, then the osteochondral border moves itself, alternatively down and up. It moves down during the hypertrophic cartilage’s growth and up during the ossification procedure. According to this modality, we were able to observe in our biopsies the presence of two osteochondral borders: one inside the hypertrophying cartilage layer and the second one located deeper due to the line of chondrocytes growing downward.

No samples were characterized by cartilage islands, typically seen in advanced active UCH. These data, showing an increased hypertrophic layer and an irregular bone–cartilage surface, suggest that the growth process and the tissue metabolisms have never really stopped; this is in line with other reports in supporting that the presence of cartilage islands is not necessarily associated with the hyperplastic growth of the mandibular condyle [38,39], and that other histological features, such as layers thickness, could be indicative of active UCH. In UCH, there is an abnormal proliferation of chondrocytes, the primary cartilage cells. These chondrocytes increase in number, leading to a thickened and expanded area of cartilage within the mandibular condyle, which could happen even without cartilage island formation.

Our data also showed an intense expression of collagen type II, MMP-2, RANK, and osteocalcin and less intense expression of collagen type I and MMP-9. 

The extracellular matrix undergoes significant changes in condylar hyperplasia, including increased production of type II collagen (characteristic of cartilage) and alterations in other matrix components like proteoglycans. These changes contribute to the rigidity and strength of the hypertrophic cartilage. Our data have shown an increased expression of collagen type II compared to type I. It is known that collagen type II is mainly present in condylar cartilage, maintaining its integrity and growth; collagen type I is also present, providing structural support and strength. Moreover, a collagen I and II switch related to the functional state of articular cartilage has been demonstrated. Our findings, showing an increased amount of collagen type II, could be explained by the biomechanical characteristics of the condyle. It is known that tensile forces are more closely associated with fibroblastic activity, which leads to the production of type I collagen. In contrast, compressive forces are correlated with chondrocytes and the increased production of type II collagen [37]. The quantity of primary collagen types (I and II) in the condylar cartilage may indicate the biomechanical and functional demands of the tissue [29]. We suggest that the increased pressure forces that usually overcome the tensile forces in UCH are responsible for activating chondrocytes to produce collagen type II.

The increase in the cartilage extracellular matrix (ECM) stimulates the activity of MMPs, such as MMP-2, specific for collagen type II [40,41], which degrades the ECM fibrillar components, allowing chondrocyte proliferation and cartilage expansion. Our data show an intense fluorescence pattern for MMP-2.

In addition, osteoclasts play an important role, the cells responsible for bone resorption, which become hyperactive in condylar hyperplasia [42]. This increased osteoclastic activity is associated with the breakdown of old bone to make space for new cartilage and bone growth. However, this process can be disordered, leading to irregular and asymmetric condylar growth. Results show many osteoclasts within Howship’s lacunae above the cartilage–bone interface. That suggests that bone metabolism is still active.

Osteocalcin is crucial in bone remodeling and mineralization and in inducing osteoclast activity [43,44]. Our data show an intense osteocalcin fluorescence pattern, particularly in the deepest layer. Cartilage infiltration into the bone is mineralized in UCH, promoting bone formation. In this way, the intense amount of osteocalcin supports the mineralization processes of cartilage needed for bone formation and condylar growth in UCH. 

The present report shows that, even if these features are moderate when compared to active UCH and are without cartilage islands, inactive UCH can be characterized by borderline features that are radiologically detectable with negative SPECT but that could represent an important trigger-point to possible reactivation, or they could represent a long slow progression. This could support Wolford, who claims that UCH is a condition that will be present at any age and is not self-limited.

Proportional condylectomy is an effective treatment option for inactive UCH, reducing the risk of reactivation of condylar growth. Some studies suggest considering conventional orthognathic surgery for inactive condylar growth or deferring surgery until the end of growth to avoid possible damage to the TMJ [20,31]. Nevertheless, proportional condylectomy has been demonstrated to have no adverse long-term effects on TMJ function when the articular disc is respected [24,45,46,47]. In this case, active rehabilitation facilitates functional recovery. Avoiding surgery in inactive cases might increase the risk of reactivation or further facial deformities, making proportional condylectomy a critical option for maintaining both aesthetic and functional outcomes in inactive UCH.

Due to the current ethical regulations, it is essential to underline the limitations of this research; a the small sample size and the impossibility of using a control group. Despite this, our data provide an original and comprehensive study that systematically describes the expression of key players of UCH, such as collagen, metalloproteinases, and bone markers like RANK and osteocalcin, alongside classic histological staining techniques in inactive UCH. 

## 5. Conclusions

Our study shows that despite the absence of cartilage islands typical of active UCH, in inactive UCH, the following mechanisms can take place: (1) increased compression forces; (2) chondrocyte activation with increased secretion of collagen type II and growth processes of the hypertrophying cartilage layer; (3) an MMP increase to digest collagen and make space for chondrocytes proliferation; (4) bone remodeling through osteoclast activation to allow cartilage invasion and, ultimately, cartilage ossification, as evidenced by osteocalcin. The present report shows that even if these features are moderate when compared to active UCH and are without cartilage islands, inactive UCH can be characterized by borderline features that may not be detectable by SPECT but that could represent an important trigger-point to possible reactivation or represent a long slow progression. That supports the hypothesis that UCH is a condition that will be present at any age and is not self-limited. Further morphological and epidemiological studies involving a larger cohort of patients will be necessary to validate our etiopathogenetic hypothesis.

Proportional condylectomy is indicated for treating inactive UCH to address facial asymmetry, occlusal disharmony, and contralateral joint dysfunction. Although growth is inactive, there remains the risk of a slow progression or reactivation. The procedure serves a curative purpose by selectively removing the overgrown condyle portion, thus correcting aesthetic and functional issues. It can also be part of a multi-step treatment, particularly when combined with orthognathic surgery or orthodontic interventions to further correct occlusal issues. By maintaining joint integrity and facilitating postoperative rehabilitation, this procedure helps prevent the risk of further facial deformities or potential reactivation of condylar growth.

## Data Availability

The data can be obtained upon request from the corresponding author. The data are not publicly accessible owing to privacy concerns.
